# Proactive clinical review of patients taking opioid medicines long term for persistent pain led by clinical pharmacists in primary care teams (PROMPPT): a non-randomised mixed methods feasibility study

**DOI:** 10.1186/s40814-025-01636-2

**Published:** 2025-04-25

**Authors:** Julie Ashworth, Nicola Cornwall, Sarah A. Harrisson, Charlotte Woodcock, Elaine Nicholls, Gillian Lancaster, Simon Wathall, Libby Laing, Toby Helliwell, Sue Jowett, Jesse Kigozi, Christian D. Mallen, Anthony Avery, Roger Knaggs, Tamar Pincus, Simon White, Clare Jinks, the PROMPPT team

**Affiliations:** 1 School of Medicine and Centre for Musculoskeletal Health Research, Keele University, Keele, Staffordshire UK; 2 Midlands Partnership University NHS Foundation Trust, Haywood Hospital, Stoke-On-Trent, Staffordshire UK; 3 Keele Clinical Trials Unit, Keele University, Keele, Staffordshire UK; 4 Synergy Primary Care Network, Nottingham, UK; 5 Health Economics Unit, Department of Applied Health Sciences, College of Medicine and Health, University of Birmingham, Birmingham, UK; 6 Centre for Academic Primary Care, School of Medicine, University of Nottingham, Nottingham, UK; 7 School of Pharmacy and Pain Centre Versus Arthritis, University of Nottingham, Nottingham, UK; 8 Primary Integrated Community Services, Nottingham, UK; 9 School of Psychology, University of Southampton, Southampton, Hampshire UK; 10 School of Pharmacy and Bioengineering, Keele University, Keele, Staffordshire UK

**Keywords:** Chronic pain, Opioids, Tapering, Acceptability, Mixed methods, Process evaluation

## Abstract

**Background:**

Given the poor long-term effectiveness of opioids for persistent non-cancer pain, and their potential for harm, evidence-based interventions to address opioid overprescribing for persistent pain are needed. This study aimed to explore the acceptability and feasibility of a primary care practice pharmacist-led intervention (PROMPPT review) for patients prescribed opioids for persistent pain and the feasibility of evaluating PROMPPT in a definitive trial.

**Methods:**

A single-arm study, with mixed methods process evaluation, was conducted in four English primary care practices. Adults prescribed opioids for ≥ 6 months were invited to participate in the Management of Opioids and Persistent Pain (MOPP) study by completing baseline and 3-month follow-up questionnaires. Practices invited a representative sample of MOPP participants to schedule a PROMPPT review, eight of which were audio-recorded. Following the review, pharmacists completed intervention delivery templates, and participants were sent an Acceptability Questionnaire and invited to consent to an interview.

**Results:**

Between November 2020 and May 2021, 148 participants were recruited to the MOPP study. Of these, 123 (83%) completed 3-month follow-up. Of 88 MOPP participants invited for a PROMPPT review, 80 (91%) attended. The review was rated completely acceptable or acceptable in 90% (45/50) of acceptability questionnaires returned. Overall, participants interviewed (*n* = 15) perceived the review as a good idea and recommended it to others; they preferred face-to-face consultations. Prior to the review, they reported mixed feelings, including feeling ‘pleased’ to be invited and ‘grateful’ someone was taking an interest, alongside concerns about what would happen during the review, including opioids being stopped and changes being detrimental. Following the review, those with a clear plan for follow-up/access to the pharmacist felt reassured about making changes to their pain medicines, whilst those advised to arrange follow-up as needed were less satisfied and more likely to report confusion about the plan.

**Conclusions:**

PROMPPT reviews appeared acceptable to patients, review uptake was high, and the study demonstrated the feasibility of a large definitive trial to evaluate PROMPPT. The review invitation, patient information, and pharmacist training were refined based on the findings ahead of a large cluster randomised controlled trial.

**Trial registration:**

ISRCTN, ISRCTN87628403, registered 31 July 2020

## Key messages regarding feasibility

- What uncertainties existed regarding feasibility?

The PROMPPT review and pharmacist training package were co-designed with stakeholders, but we did not know how acceptable the review would be to patients, how feasible it would be for practice pharmacists to deliver, or whether the training would enable them to do so as intended. There were also uncertainties around the feasibility of recruiting the target population from general practice, retaining participants at follow-up, and the suitability of data-collection procedures to evaluate the intervention in a definitive trial.

- What are the key feasibility findings?

This study demonstrated the feasibility of practice pharmacists delivering PROMPPT reviews in primary care. Review uptake was high, and the review appeared acceptable to patients. The study successfully recruited the target population and retained 80% at 3-month follow-up. Completion rates of key outcome measures in the self-reported questionnaires were high, which gives confidence in the use of these measures in a main trial.

- What are the implications of the feasibility findings for the design of the main study?

The PROMPPT intervention and proposed cluster randomised controlled trial to evaluate PROMPPT were concluded to be feasible with only minor adjustments. We refined the trial recruitment process, PROMPPT review invitation, patient information resources, and pharmacist training based on the findings. Audio-recorded consultations yielded valuable information to create case studies for the training. We also refined the pain medicines and healthcare resource use questionnaires to optimise data collection.

## Background

Opioids are commonly prescribed for persistent non-cancer pain (persistent pain) [1, 2], despite poor long-term efficacy and increased risk of serious harm [3–5]. Consequently, reducing inappropriate opioid use is a key healthcare priority in many countries, including the United Kingdom (UK). However, evidence supporting interventions to reduce opioid use is sparse and often of low methodological quality [6, 7]. A Cochrane review identified the need for further, larger randomised controlled trials (RCTs) of theoretically grounded behaviour change interventions focussing on reducing opioids in the context of persistent pain [6]. Sandhu et al. [8] subsequently evaluated a community-based multicomponent intervention consisting of group meetings, education, individual support, and skill-based learning to reduce opioid use. This RCT found that among patients with chronic pain due to non-malignant causes, a group-based educational intervention significantly reduced opioid use compared with usual care and had no effect on perceived pain interference with daily activities [8]. However, less than half of participants randomised to the group intervention attended all sessions. Given the diversity of patients who are prescribed opioids for persistent pain, a range of evidence-based strategies are likely to be needed [9].

One strategy, recommended in best practice guidelines, is regular review of patients taking opioids for persistent pain to assess the balance of benefit versus potential harm and gradual tapering of opioids if treatment goals are not met [10]. However, most long-term opioid prescribing occurs in primary care, and, in the United Kingdom (UK), the time available in general practitioner (GP) appointments offers limited opportunity to routinely conduct comprehensive opioid reviews. To address challenges facing UK primary care [11], there has been an expansion in the number of clinical pharmacists working in GP practices to help manage patients on long-term medicines [12]. These ‘practice pharmacists’ conduct face-to-face consultations with patients in GP surgeries. A systematic review assessed the effectiveness of pharmacist-delivered interventions to optimise opioid therapy and improve care for people with persistent pain, identifying potential benefits across a range of settings, and recommended further research to guide the expansion of pharmacist roles in this area [13].

This study is the second phase of a programme of research to develop and test a practice pharmacist-led primary care intervention for patients prescribed long-term opioids for persistent pain called PROMPPT (*P*roactive clinical *R*eview of patients taking *O*pioid *M*edicines long term for persistent *P*ain led by clinical *P*harmacists in primary care *T*eams). In line with Medical Research Council (MRC) guidance on the development and evaluation of complex interventions, the PROMPPT intervention was co-designed with stakeholders (patients and healthcare professionals), using a person-based approach [14] combined with best practice guidance and theory on behaviour change. Extensive intervention development work in the first phase included qualitative interviews with patients, clinical pharmacists and GPs, an online qualitative study, and in-practice testing of prototype PROMPPT consultations with patients and practice pharmacists in three GP practices [15–17]. Findings informed refinement of the intervention components and content of the pharmacist training programme ahead of this feasibility study.

### Aims and objectives

The aims of this study were to assess the acceptability and feasibility of PROMPPT and the study methods to evaluate PROMPPT in a future definitive trial.

Specific objectives were as follows:

1. Investigate rates of recruitment and retention of patient participants in the Management of Opioids and Persistent Pain (MOPP) questionnaire study.
2. Examine the completeness and suitability of data collection to evaluate clinical and cost-effectiveness in a future cluster RCT.
3. Investigate intervention uptake by examining what proportion of MOPP study participants invited scheduled and attended a PROMPPT review.
4. Investigate the acceptability and credibility of the PROMPPT review.
5. Examine the fidelity of PROMPPT review delivery by practice pharmacists.

## Methods

### Study design

A single-arm, mixed methods design, delivered in four general practices, was used to meet the study objectives. Mixed methods were used to understand if, how, and why or why not the PROMPPT review was delivered and engaged with, thereby allowing for a better understanding of feasibility ahead of a large-scale RCT than if either quantitative or qualitative data had been collected alone. As Keele Clinical Trials Unit (CTU) and the study team had extensive experience of conducting cluster RCTs in UK primary care which have recruited patients with musculoskeletal pain [18–20], we did not identify any uncertainties to address that required a control arm. Nevertheless, the extension of CONSORT 2010 checklist for pilot and feasibility randomised trials [21] has been used, where applicable, in reporting. Ethical approval for the study was granted by the UK Health Research Authority (Research Ethics Committee Reference: 20/NS/0067). The study was registered on the ISRCTN Registry (ISRCTN87628403), and the study protocol is available at https://www.isrctn.com/ISRCTN87628403.

### Participant eligibility and identification

The target population was adults prescribed an opioid medicine for persistent non-cancer pain. Potentially eligible patients were identified from electronic medical records (EMRs) and grouped according to opioid strength prescribed (weak, intermediate, strong — see Table 1) based on a published primary care categorisation of prescribed analgesics [22]. Eligibility was confirmed by GP screening. Inclusion criteria were as follows: aged 18 years or over and prescribed any opioid or opioid/paracetamol combination from Sects. 4.7.2 and 4.7.1 British National Formulary [23] for at least 6 months, with a prescription issued within the previous 2 months. Exclusions were acute pain, cancer pain, terminal illness (life expectancy less than 6 months), vulnerable patients (e.g. severe mental illness, learning difficulties, dementia), current treatment for substance misuse, and inability to understand English.

**Table 1:** Categorisation of patients by opioid strength^a^

Weak	Intermediate	Strong
Co-codamol 8 mg/500 mg	Codeine 30 mg	Morphine
Co-codamol 15/500 mg	Co-codamol 30 mg/500 mg	Oxycodone
Codeine 15 mg	Dihydrocodeine 30 mg	Fentanyl
Codeine 20 mg	Buprenorphine patch ≥ 1 5 mcg/h	Tapentadol
Co-dydramol 10 mg/500 mg	Buprenorphine SL 400 mcg	Diamorphine
Co-dydramol 20 mg/500 mg	Tramadol > 3 7.5 mg	Hydromorphone
Dihydrocodeine 20 mg	Pethidine	Dipipanone
Co-proxamol 32.5 mg/325 mg	Pentazocine	Dextromoramide
Tramadol 37.5 mg/500 mg	Meptazinol	
Buprenorphine patch 5 or 10 mcg/h		
Buprenorphine sublingual 200 mcg		

^a^Hierarchy of analgesic potency arising from a consensus study of UK general practitioners [22]

### Recruitment

#### General practices

The UK National Institute for Health and Care Research (NIHR) Clinical Research Networks (CRNs) facilitated recruitment of four general practices, two in the West Midlands region and two in East Midlands, aiming for practices that varied in location (urban, semi- urban, rural) and population socio-demographics. Due to the uncertainty about the impact of the COVID-19 pandemic on recruitment, and to ensure adequate numbers of participants on high strength opioids, practices were eligible if they had at least 10,000 registered patients and had a practice pharmacist, who was an independent prescriber, working in the practice for at least one session per week. Consent for observation/audio-recording of a sample of PROMPPT consultations (with patient consent, aiming for two per practice) was obtained from the practice pharmacist in each participating practice.

#### Patients

Participating practices invited eligible patients to consent to be contacted about the MOPP questionnaire study. Those who returned a completed consent-to-contact form to Keele CTU were mailed a MOPP study recruitment pack, with reminders sent to nonresponders after 2 and 4 weeks. Consent was provided by potential participants returning a completed baseline questionnaire and consent form. Potential participants were also invited to consent to the research team accessing a depersonalised download from their electronic medical record and contact about future related research.

MOPP study participants who consented to contact about related research and subsequently scheduled a PROMPPT review were invited to consent to observation and/or audio-recording of their initial PROMPPT review consultation. Following the consultation, these participants were also mailed a process evaluation recruitment pack by Keele CTU, inviting them to complete and return an Acceptability Questionnaire and to take part in an interview. Participants could consent to any, both, or neither of these options. Participants who consented to further contact about an interview were contacted by a researcher to arrange a mutually convenient time and location for this. Once the target sample size (*n* = 15) for participant interviews had been reached, subsequent participants returning an interview reply form were sent a letter thanking them for their interest and advising them that they would not be invited to participate.

### Intervention

PROMPPT is a primary care intervention comprising a proactive practice pharmacist-led review for patients who have been prescribed long-term opioids for persistent non-cancer pain and an associated pharmacist training package. PROMPPT aims to reduce opioid use, where this is appropriate, without increasing pain or pain-related interference.

### PROMPPT review

PROMPPT review invitation letters, sent by Keele CTU on behalf of the GP practices, invited MOPP study participants to contact the practice to schedule a 30-min appointment for an initial consultation with the practice pharmacist, conducted face-to-face or remotely (by video or telephone) according to the prevailing social distancing measures due to the COVID-19 pandemic and participant preference. The invitation was accompanied by a ‘Pain Concerns Form’, which was developed in conjunction with stakeholders (patients and healthcare professionals) from Pain Concern’s Pain Navigator Tool [24]. The aim of the Pain Concerns Form was to help focus the review consultation on what was most important to the patient. Participants were asked to complete the form, prior to their appointment, by reading a list of statements representing common concerns reported by people with persistent pain, ticking those statements they agreed with, and adding any further concerns or comments in a free text box.

In brief, the initial PROMPPT review consultation comprised a holistic assessment of the participant’s experience of persistent pain and the impact of pain on daily life; a personalised discussion to explore their experience of the effects (wanted/unwanted, useful/bothersome) of opioids; an exploration, using motivational interviewing techniques, of their readiness to change their opioid medicines; identifying and addressing important barriers to reducing opioids specific to the individual, such as fear of pain worsening and/or withdrawal symptoms; and an individualised management plan, agreed using a shared decision-making approach. Opioid tapering was not mandatory, for example, if a participant continued to obtain functional benefit from moderate dose opioids, without experiencing troublesome side effects. Management plans could also include advice and information leaflets relating to self-management, signposting to online information resources, and signposting or referral to appropriate community services (for example physiotherapy, exercise classes, and community psychology services) and, for more complex cases, discussion/collaboration with the GP and/or referral to specialist services if needed. Shorter follow-up appointments (up to 15 min) were arranged according to clinical need, face-to-face or remotely by video or telephone, according to social distancing requirements and patient preference. The practice pharmacists delivering PROMPPT reviews were all independent prescribers and had completed specific training to deliver the PROMPPT intervention, as described below.

### Pharmacist training

A training package to enable practice pharmacists to deliver the PROMPPT pain reviews was developed by experts in pain management, primary care clinicians, behaviour change experts, and medical/pharmacy educationalists. The training comprised an e-learning course, delivered via the PROMPPT study website, a training manual (printed and pdf versions available), and two half-day online workshops delivered via Microsoft Teams.

Training content was informed by findings from earlier intervention development work [15–17]. The training covered communication skills, communication of risk and benefit in personalised discussions about opioids, motivational interviewing techniques, negotiating treatment plans, creating opioid tapering plans, optimising non-opioid pain management, supporting self-management for persistent pain, signposting to patient information resources, and when to seek help (e.g. from a GP). Training included self-care strategies to help practice pharmacists manage any emotional impact of conducting pain reviews. Practice pharmacists were also provided with study-specific training including completion of study documentation, good clinical practice as applicable to research, adverse event reporting, and maintenance of study records.

The online workshops were facilitated by members of the research team with clinical and academic expertise in persistent pain management and behaviour change, supported by two clinical champions, who were experienced clinical pharmacists and had completed the PROMPPT e-learning and received additional training in mentoring and providing feedback. The online workshops incorporated role play, in simulated consultations, with feedback from the facilitators. In addition, practice pharmacists delivering PROMPPT reviews were mentored during the study by one of the clinical champions.

### Feasibility outcomes/data collection

An overview of data collection methods for each feasibility objective, pre-specified progression criteria, and outcomes reported is provided in Table 2.

**Table 2:** Feasibility objectives, progression criteria, data collection methods, and outcomes reported

	**Feasibility objectives**	**Questions to be answered**	**Pre-specified progression criteria**	**Quantitative methods**	**Qualitative methods**	**Outcomes reported**
1	Evaluation of recruitment and retention	Can we recruit and retain eligible patient participants in the questionnaire study?	≥ 20% of eligible patients consent to participate ≥ 70% of participants complete 3-month follow-up questionnaire	Study flow, self-reported participant questionnaires		Flowchart, recruitment and retention rates, participant characteristics
2	Evaluation of participant data collection	How appropriate are the data collection procedures to evaluate the intervention in the intended population?	N/A	Self-reported participant questionnaires, prescribing data from electronic primary care record	Semi-structured interviews with participants	• Completion rates/missing data • Qualitative evaluation of data collection methods
3	Evaluation of intervention uptake	What proportion of those invited schedule and attend a PROMPPT review?	> 50% of MOPP participants invited to attend at least one PROMPPT review consultation	Study flow	Semi-structured interviews with participants	• Rates of intervention uptake by eligible participants • Qualitative evaluation of acceptability according to TFA domains
4	Evaluation of intervention acceptability and credibility	Is the intervention credible and acceptable to patient participants?	• Mean acceptability/credibility score ≥ 5 • Evidence from interviews about intervention acceptability	Acceptability questionnaire^a^	Semi-structured interviews with participants	• Acceptability questionnaire scores • Qualitative evaluation of acceptability according to TFA domains
5	Evaluation of the fidelity of intervention delivery	Do practice pharmacists deliver the intervention as the training intended?	N/A	Practice pharmacist-complete case report forms (CRFs)	• Audio-recorded consultations • Semi-structured interviews	Delivery of key intervention components as per protocol

^a^Acceptability questionnaire comprised of the theoretical framework of acceptability (TFA, items 1–8 scored on 0–5 scale) [25] and a modified acceptability and credibility measure (items 9–12, scored on 0–10 scale) derived from an existing measure [26]

#### Recruitment and retention

We examined the numbers of potentially eligible patients identified from EMR searches, the numbers of eligible patients (after screening by GPs) invited, the number and characteristics of those who consented to participate, the number and characteristics of participants who returned a completed 3-month follow-up questionnaire and estimated the proportion of eligible patients who consented to participate, and the proportion of participants retained at 3-month follow-up.

#### Participant data collection

Participants were asked to complete and return self-report postal questionnaires that included the proposed primary and secondary outcome measures for a future definitive trial. The proposed co-primary outcomes were as follows: the Brief Pain Inventory (BPI), a measure of pain severity and pain-related interference [27], and reduction in opioid use (expressed as reduction in daily morphine equivalent dose (MED)), reflecting the importance to patients, confirmed by our PPI group, that pain and pain-related interference does not increase when reducing opioids. We explored the feasibility of calculating daily MED using two different data sources: self-reported questionnaire data and electronic prescribing records. A pain medicines use questionnaire (developed for this study) collected information on dose and frequency of use for each opioid and non-opioid pain medicines used during the preceding 4 weeks. Electronic prescribing data was extracted for a period starting 56 days prior to the date the first participant in a practice was recruited to the date of the final 3-month follow-up questionnaire and was securely transferred to Keele CTU.

Participant self-reported questionnaires also included the following measures: medication-related side-effects using a checklist derived from a previous study [28], the Pain Self-Efficacy Questionnaire [29], and health-related quality of life using the EQ-5D-5L [30]. Finally, a bespoke healthcare resource use questionnaire, administered at follow-up, assessed participants’ use of National Health Service (NHS) and private health care, as well as over-the-counter purchases, focusing on healthcare resource use related to persistent pain and/or opioid-related side effects. This questionnaire also requested information on time off work, occupation, typical work activities and the nature of participants’ employment (full time or part time), and the single-item presenteeism question from the Work Productivity and Activity Impairment Questionnaire [31, 32] was used to estimate presenteeism.

#### Intervention uptake

We examined the numbers of participants invited to schedule a PROMPPT review and the number and characteristics of those who scheduled and attended at least one PROMPPT consultation. To assess intervention (PROMPPT review) uptake, we calculated the proportion of participants invited who attended the initial PROMPPT consultation.

#### Acceptability and credibility of the PROMPPT review

Mixed methods data were collected from participant interviews and an Acceptability Questionnaire, which was mailed to all participants who attended a PROMPPT review. The Acceptability Questionnaire comprised 12 questions from 2 existing measures: the theoretical framework of acceptability (TFA) [25] and a modified version of a treatment acceptability and credibility measure [26], which has been used in previous similar studies. The TFA questionnaire [25] comprises eight items (scored on a 1–5 scale). The first is a global acceptability question, and the remaining seven represent key constructs of acceptability relating to healthcare interventions (affective attitude, burden, intervention coherence, ethicality, perceived expectations, opportunity cost, and self-efficacy). The acceptability and credibility measure includes four items (scored on a 0–10 scale) and assesses (1) how logical the PROMPPT review seems to participants, (2) how confident participants are that it will be successful in helping them, (3) how confident participants would be in recommending it to a friend, and (4) how satisfied they were with the intervention overall.

Semi-structured interviews were conducted with 15 participants (sampled from across the 4 participating practices). Interview topic guides were informed by patient stakeholders and by programme theories regarding behaviour change, implementation, and acceptability [25, 33–35]. Participants were interviewed after attending at least one appointment for a PROMPPT review with the practice pharmacist. All interviews were conducted by an experienced qualitative researcher and, with participants’ consent, were audio recorded. The interview audio-recordings were transcribed verbatim by a professional transcription company and were anonymised by members of the research team ahead of analysis.

#### Fidelity of intervention delivery in practice

Fidelity of PROMPPT review delivery was assessed using evidence from audio-recordings of PROMPPT reviews, case report forms (CRFs), and participant interviews. A sample of eight consultations were digitally audio-recorded, with pharmacist and participant consent, and practice pharmacists were asked to complete a CRF, which included an intervention delivery template, after each PROMPPT consultation. Data from audio-recordings and CRFs were assessed against a pre-specified checklist to determine delivery of eight key intervention components. These were as follows: (1) inviting the participant to tell their pain story, (2) using the Pain Concerns Form, (3) exploring the effects of opioids, (4) assessing the participant’s perspective on changing opioids, (5) discussing self-management for persistent pain, (6) agreeing a management plan, (7) providing a pain review plan and information resources, and (8) discussing follow-up and further contact arrangements.

### Sample size

Based on an assumption that 25% of patients invited would consent to join the study, we determined that a sample of 80 eligible participants would allow us to estimate the overall consent rate with precision (defined as half the width of a 95% confidence interval) of at least ± 10%. Similarly, if the 3-month follow-up rate was around 75%, this estimate would have precision of around ± 10%. Based on data from previous unpublished analysis of our regional anonymised primary care database (Consultations in Primary Care Archive (CiPCA)), we expected to identify around 270 patients with persistent pain prescribed long-term opioids in a GP practice with around 5000–6000 patients. Assuming GP screening excluded 30% of patients identified and a minimum of 25% of those contacted consented to participate, we estimated a mean of at least 47 eligible patients per practice of this size would consent to participate. However, we chose to recruit from four general practices to test recruitment, retention, and intervention delivery across a range of settings and included practices with at least 10,000 patients due to uncertainty about the impact of the COVID-19 pandemic on recruitment, acknowledging that this might yield more participants than the number (*n* = 80) we had planned for. In this event, a sample of around 20 MOPP participants per practice, selected to reflect the approximate opioid group distribution (weak, intermediate, strong) within the practice overall, were invited for a PROMPPT review.

### Data analysis

#### Quantitative data analysis

Participant baseline characteristics were described (using means, standard deviations, medians, interquartile range, numbers, and percentages as appropriate) to characterise the sample and highlight any potential areas of selection bias that could be minimised in a main trial design. Participants’ baseline characteristics were explored in the following subgroups, but between-group differences were not tested for statistical significance as per the CONSORT extension guidelines for feasibility studies [21]:

- Eligible patients mailed a study invitation (date of birth, sex, and opioid group)
- Eligible patients consenting to participate and returning a baseline questionnaire
- Participants returning a 3-month follow-up questionnaire

Missing data rates at baseline were calculated for each outcome measure in the self-report questionnaire, and the level of completion and suitability of the resource use questionnaire, administered at 3-month follow-up, was assessed. Baseline pain medicines data from self-report questionnaires and electronic prescribing records were used, where possible, to calculate daily MED. A MED calculator was created in Microsoft Excel (2016) by lead author JA using published opioid conversion factors, as shown in Table 3 [36–38], and refined following testing with members of the research team (SAH, TH, SW, RK.). Information on opioid name, strength, dose, and frequency of use was extracted separately from self-report questionnaires and electronic prescribing records and entered into the MED calculator by clinical academics from the research team (JA, SAH, TH, SW, DA.).

**Table 3:** Morphine equivalent conversion factors

	**Conversion factor**^**a**^	**Equivalent dose to 10-mg morphine**	
**Oral preparations (mg/day)**			
Codeine	0.1		100 mg
Dihydrocodeine	0.1		100 mg
Dextropropoxyphene	0.23^b^		43.5 mg
Tramadol	0.1		100 mg
Tapentadol	0.4		25 mg
Oxycodone	1.5		6.6 mg
Pethidine (meperidine)	0.1^b^		100 mg
Hydromorphone	5.0		2 mg
**Sublingual preparations (mg/day)**			
Buprenorphine	40^c^		0.25 mg
**Transdermal patches (micrograms/hour)**			**Morphine equivalent (mg/day)**
Buprenorphine (5)	2.4		12
Buprenorphine (10)	2.4		24
Buprenorphine (20)	2.4		48
Fentanyl (12)	2.5		30
Fentanyl (25)	2.4		60
Fentanyl (50)	2.4		120
Fentanyl (75)	2.4		180
Fentanyl (100)	2.4		240

^a^Conversion factors are approximations of equianalgesic doses, and there is some variation across different sources. The conversion factors used in this study are those accepted by the UK Faculty of Pain Medicine, Royal College of Anaesthetists (Opioid Aware (37)), except for dextropropoxyphene, pethidine, and sublingual buprenorphine, which are not included in the Opioid Aware list. The source of conversion factors for these drugs is below

^b^CONSORT morphine equivalent conversion factors (36) (dextropropoxyphene, pethidine)

^c^Faculty of Pain Medicine, Australian and New Zealand College of Anaesthetists (ANZCA) (38) (sublingual buprenorphine)

Data were also extracted regarding use (yes or no) of the following classes of non-opioid pain medicines: paracetamol, topical treatments, nefopam, systematic NSAIDs, gabapentinoids, antidepressants, benzodiazepines, and Z-drug sleeping tablets. The results from the two data sources were compared.

The mean and standard deviation were calculated for the intervention acceptability/credibility measure (items 9–12 of the Acceptability Questionnaire). A mean score ≥ 5/10 for each item was the threshold for acceptability/credibility, as in the previous trials using this measure [39, 40]. TFA responses (questions 1–8 of the Acceptability Questionnaire) were summarised descriptively.

#### Qualitative data analysis

A framework approach [41] was used for qualitative data analysis, consisting of familiarisation with and coding of transcripts, summarising data, entering data into a matrix, and identifying detected elements within summarised data [41, 42]. Research team members from different professional backgrounds (including GPs and clinical pharmacists) discussed data to increase trustworthiness of the analysis [43]. Management of qualitative data during analysis was facilitated using QSR NVivo version 12 [44]. Continuous team discussion helped to challenge interpretations of data and refine coding according to theoretical constructs. This framework approach allowed for both deductive (a priori) themes based on the TFA [25] and inductive themes to be identified.

#### Mixed methods analysis

Integrated data analysis was undertaken to merge data about acceptability and fidelity from quantitative and qualitative sources. Statistics-by-themes joint displays were generated to compare results, identify similarities and differences in datasets, and develop meta-inferences.

### Patient and public involvement (PPI)

Patient contributors from the Research Users Group at Keele University (*n* = 5), who were familiar with the development of the PROMPPT intervention, were invited to join a PPI group for this study. The aims of PPI were as follows: (1) To advise on practicalities of delivering the study from a patient perspective; (2) to help interpret the study findings from a patient perspective, including providing their perspective on themes from the qualitative data analysis; and (3) to advise on how the intervention and design of a future main trial could be revised, in light of these findings, to make it more acceptable/appealing to patients. Patient contributors were supported by a dedicated PPI coordinator and user support worker and reimbursed for their time and costs.

The PPI group convened for online workshops on two occasions. A written summary of the discussion and outcomes was sent to all members of the group following each meeting. The first workshop (August 2020, prior to the study commencing) discussed the potential impact of COVID-19 and the possibility of delivering PROMPPT reviews remotely. The group advised that due to prevailing social distancing measures, remote reviews would be acceptable but advised offering choice and flexibility, where possible, including how patient-facing documents were shared (paper copy or electronically), providing clear instructions about what would happen and a back-up plan in case technological difficulties arose during the consultation. The group also recommended exploring how well remote consultations worked in the interviews. The study protocol and interview topic guides were amended accordingly. The second PPI workshop was held in July 2021, to discuss study findings and how these could be used to refine the intervention.

## Results

Nine eligible GP practices were approached about the study. Five expressed an interest in participating and agreed to conduct feasibility searches of their EMRs to estimate the number of potentially eligible patients. One practice had insufficient numbers of patients on opioids and was excluded. Four eligible GP practices (two West Midlands, two East Midlands) were recruited with a total of *n* = 55,346 registered patients.

### Objective 1: Participant recruitment and retention

**Fig. 1:**
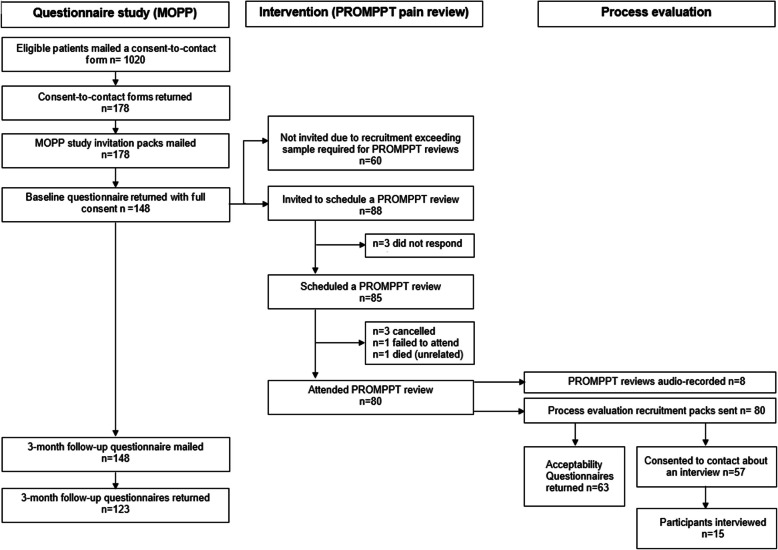
Study flowchart

Searches of the EMRs of the four participating practices identified 1748 potentially eligible patients. One practice (list size 19,244) identified substantially more potentially eligible patients (*n* = 742) than the others and had the list for GP screening reduced to 300, keeping the distribution of opioid strength approximately proportionate to the distribution in the full list. GP screening confirmed 1020 eligible patients who were invited to consent to be contacted about the study. Between 26 November 2020 and 20 March 2021, 148 participants were recruited (see Fig. 1). The proportion of eligible patients who consented and returned a baseline questionnaire was somewhat lower (14.5%) than the pre-specified success criterion of ≥ 20%, but retention at 3-month follow-up was higher at 80% (target ≥ 70%).

The study population was 64% female, with a mean (SD) age of 67 (12.9) years at baseline (see Table 4). Baseline characteristics for those who returned a completed 3-month follow-up questionnaire were broadly similar except that the proportion of participants in the strong opioid group was lower at 9% versus 12%, and daily morphine equivalent dose (MED) was slightly lower (mean 25.98, *SD* 25.91 versus mean 27.95, *SD* 28.75).

**Table 4:** Participant baseline demographics and pain-related clinical characteristics

Baseline characteristic	All participants (*n* = 148)		Participants returning a 3-month questionnaire (*n* = 123)		Participants attending PROMPPT review (*n* = 80)	
	Missing data *n* (%)	Descriptive statistic	Missing data *n* (%)	Descriptive statistic	Missing data *n* (%)	Descriptive statistic
Age: Mean (SD)	0 (0)	67 (12.9)	0 (0)	68 (12.4)	0 (0)	69 (12.0)
Female sex: *n* (%)	0 (0)	95 (64)	0 (0)	77 (63)	0 (0)	47 (59)
Opioid group: *n* (%)	0 (0)		0 (0)		0 (0)	
Weak		33 (22)		29 (24)		20 (25)
Intermediate		97 (66)		83 (67)		49 (61)
Strong		18 (12)		11 (9)		11 (14)
Length of time with persistent pain: *n* (%)	1 (1)		1 (1)		0 (0)	
< 1 year		1 (1)		0 (0)		0 (0)
1–2 years		4 (3)		2 (2)		1 (1)
3–5 years		25 (17)		22 (18)		11 (14)
6–10 years		38 (26)		35 (29)		22 (28)
> 10 years		79 (54)		63 (52)		46 (58)
Brief Pain Inventory: Total score (0–10): mean (SD)	4 (3)	5.8 (1.9)	3 (2)	5.9 (1.8)	0 (0)	5.7 (1.9)
Pain severity (0–10): mean (SD)	4 (3)	5.6 (1.9)	3 (2)	5.6 (1.8)	0 (0)	5.4 (1.9)
Pain interference (0–10): mean (SD)	0 (0)	6.2 (2.3)	0 (0)	6.1 (2.2)	0 (0)	6.0 (2.3)
Pain Self-Efficacy Questionnaire (0–54^a^): mean (SD)	8 (6)	26.4 (11)	6 (4)	26.8 (10.7)	2 (3)	28.1 (11.2)
Morphine equivalent dose^b^: mean (SD)	14 (9)	27.95 (28.75)	11 (9)	25.98 (25.91)	6 (8)	27.67 (28.75)
Morphine equivalent dose^b^: median (IQR)	14 (9)	19.40 (12.00, 31.23)	11 (9)	18.00 (12.00, 30.00)	6 (8)	18.55 (11.30, 32.00)
Non-opioid pain medicines used for pain: *n* (%)	0 (0)	137 (93)	0 (0)	112 (91)	0 (0)	74 (93)
None		11 (7)		11 (9)		6 (8)
Paracetamol		122 (82)		102 (83)		69 (86)
Topical treatments		6 (4)		5 (4)		4 (5)
Nefopam		1 (1)		1 (1)		0 (0)
Systematic NSAIDs		27 (18)		23 (19)		13 (16)
Gabapentinoids		22 (15)		17 (14)		12 (15)
Antidepressants		23 (16)		17 (14)		10 (13)
Benzodiazepines		0 (0)		0 (0)		0 (0)
Z-drugs		2 (1)		0 (0)		2 (3)
Health-related quality of life: EQ-5D-5L (− 0.594 to 1): Mean (SD)	9 (6)	0.50 (0.23)	3 (4)	0.53 (0.20)	3 (4)	0.53 (0.20)

^a^The pain self-efficacy questionnaire comprises 10 questions measured on a 0 to 6 scale leading to a score range of 0 to 60. Our score only ranges from 0 to 54 as there was an error in the wording of one of the questions on our questionnaires so it could not be included in the data to calculate a total score. This error will be corrected prior to the main study commencing. *SD* standard deviation, *IQR* interquartile range

^b^Morphine equivalent dose calculated from self-reported questionnaire data alone for 126 participants and self-reported data, supplemented by information regarding drug name and/or strength from the EMR where there were incomplete/ambiguous entries (8 participants)

### Objective 2: Completeness and suitability of data collection

**Fig. 2:**
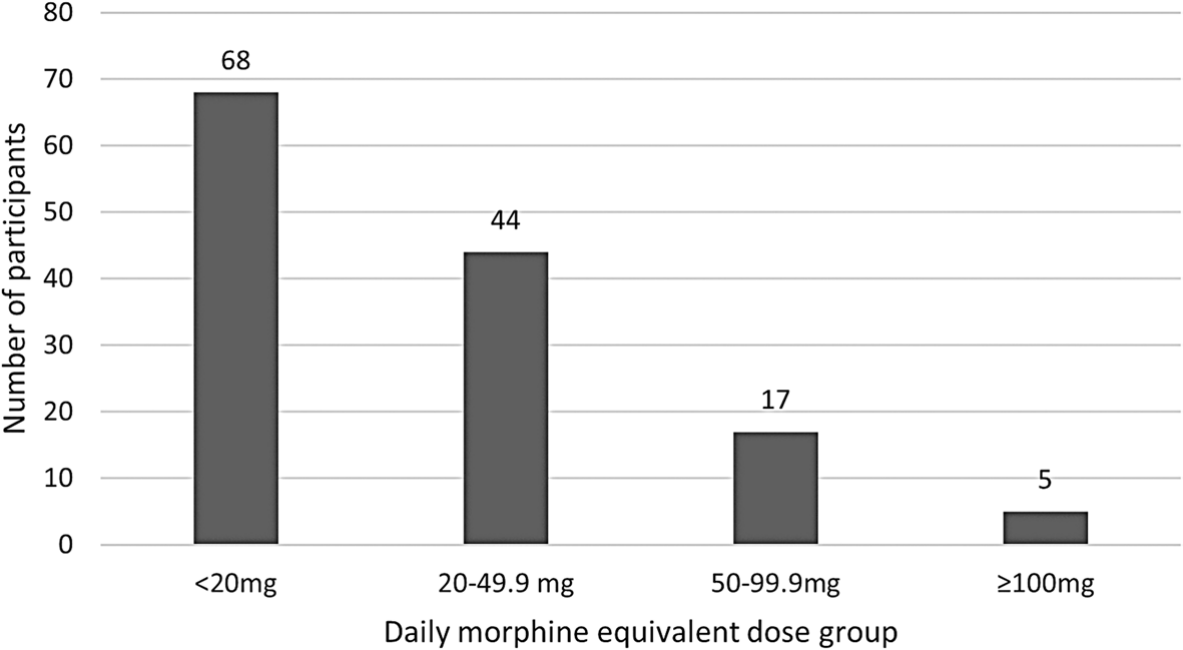
Participants’ baseline opioid use categorised by average daily morphine equivalent dose

Self-reported pain medicines use questionnaires included sufficient information to calculate daily MED in 126 out of 148 cases at baseline. In cases where incomplete or ambiguous entries precluded MED calculation from self-reported data alone (*n* = 8 participants), we were able to obtain the missing information regarding drug name and/or strength from participants’ EMRs, increasing the total to 134 out of 148 (91%) cases. Average daily MED for these participants, categorised according to dose (< 20 mg, 20–49.9 mg, 50–99.9 mg, and ≥ 100 mg [45]), is shown in Fig. 2.

Using electronic prescribing data alone, we were only able to extract sufficient information to calculate baseline daily MED in 72 out of 148 cases, all of whom were registered with two practices using the EMIS Web EMR system [46]. The other two practices (68 participants) used the SystmOne EMR system [47], which did not allow the research team to extract sufficiently detailed information to calculate the MED. The SystmOne download only included name and strength of each drug, whereas the EMIS download included dose, frequency, prescribing instructions, and amount issued. The remaining eight participants did not have an opioid issued during the pre-specified baseline period (56 days prior to the date of baseline questionnaire completion), despite their questionnaire indicating that they had used opioids at baseline. It seems likely that these participants used opioids on an ‘as required’ basis and requested prescriptions less frequently than every 56 days. Mean (SD) daily morphine equivalent dose (MED), calculated from self-reported questionnaire data alone (*n* = 126), was 28.62 (29.15) mg, but the distribution was skewed, with a few participants having very high MED, so the median (IQR) was lower at 19.85 (12.00, 31.88) mg. When self-reported questionnaire data were supplemented by EMR data, where needed (*n* = 134), the results were similar: mean (SD) MED 27.95 (28.75) mg and median (IQR) 19.40 (12.00, 31.23) mg. Daily MED calculated from electronic prescribing records (*n* = 77) was lower: mean (SD) 16.84 (13.57) mg and median (IQR) 12.00 (7.18, 21.40) mg. In part, this may be due to assumptions made during the calculations because, for 34/77 (44%), it was not possible to calculate daily MED from the electronic prescribing instructions because these allowed for a range of dose and/or frequency of use ‘as required’. In these cases, daily MED was calculated by dividing the amount supplied by the number days in the supply period. When MED results and other pain medicines use from self-reported data were compared with those from electronic prescribing records, for individual participants (*n* = 64) with results from both data sources, we found that MED from self-reported data was greater or equal to MED from prescribing data in 47/64 (73%) of cases. Self-reported use of non-opioid pain medicines (paracetamol, topical treatments, nefopam, NSAIDs, gabapentinoids, antidepressants, benzodiazepines, and Z-drug sleeping tablets) was identical to the electronic prescribing record for 41 participants (51%), and 11 participants recorded additional non-opioid pain medicines (paracetamol and/or NSAIDs) which were available ‘over the counter' without prescription. The electronic prescribing records of a further 11 participants included an antidepressant when the participant had not recorded this on their pain medicines use questionnaire, but the reason for prescribing is not included in the EMR and may not have been perceived to be related to pain. In the remaining 17 cases where differences occurred, these tended to be minor discrepancies, for example topical NSAID gels prescribed on the medical record but not included in the self-report data.

Completion rates of other key outcome measures in the self-reported baseline questionnaires were high (> 97% for the BPI, > 94% for the Pain Self-Efficacy Questionnaire, and > 93% for the EQ-5D-5L). The self-reported health resource use questionnaire at 3-month follow-up showed good completion rates with minimal errors and missing data. For the initial yes/no filter for resource use questions, completion rates were 86 to 94%, with remaining responses left blank; this was highest for questions on visits to healthcare professionals (*n* = 19, 15%). However, this did not prevent interpretation of the response, as in some cases further details were still given, or for the majority, no items chosen. Within-question completion rates (giving further details if ‘yes’ was chosen) were between 92 and 100%. The filter question on being in a paid job was completed by > 97% of respondents.

### Objective 3: Intervention uptake

As anticipated, more MOPP participants (*n* = 148) were recruited than required so a representative sample of around 20 MOPP participants per practice, selected to reflect the approximate distribution of opioid groups (weak, intermediate, strong) within the practice overall, were invited for a PROMPPT review. Of 88 participants invited, 85 (97%) scheduled a PROMPPT review, and 80 (91%) attended the initial PROMPPT consultation (see Fig. 1). This exceeded the pre-specified success criterion of > 50% of those invited attending at least one PROMPPT consultation. The baseline characteristics of participants attending a PROMPPT review (see Table 4) are broadly similar to MOPP participants overall, although the proportion of females is somewhat lower at 59% (versus 64%).

### Objective 4: Acceptability and credibility of the PROMPPT review

#### Quantitative evaluation

Of the participants who attended a PROMPPT review, 63/80 returned a completed Acceptability Questionnaire (see Table 5). The TFA questions (items 1–8) showed that the PROMPPT review was acceptable to most participants (90%) and required little effort to participate (88%). Most participants understood how the review could help them to manage their pain (66%), with 60% believing that by attending a PROMPPT review they were likely to change how they manage their pain. The mean acceptability/credibility score (items 9–12) was 6.67 (*SD* 2.20), which exceeded the pre-specified success criterion of ≥ 5.

**Table 5:** Acceptability questionnaire

		*n* (%)
**Theoretical framework of acceptability (TFA)**		*n* = 63
1. How acceptable was the pain management review?	Acceptable or completely acceptable	45 (90%)
2. Did you like or dislike the pain management review?	Like or strongly like	38 (76%)
3. How much effort did it take to participate in the pain management review?	No effort at all or A little effort	44 (88%)
4. How fair (to all patients) is a system where patients with long-term pain are invited for a routine pain management review?	Fair or very fair	42 (84%)
5. The pain management review is likely to change how I manage my pain	Agree or strongly agree	30 (60%)
6. It is clear to me how the pain management review I attended with the clinical pharmacist will help me manage my pain	Agree or strongly agree	33 (66%)
7. How confident would you feel about making changes to how you manage your pain?	Confident or very confident	34 (68%)
8. Making changes to how I manage my pain will interfere with my other priorities	Strongly disagree or disagree No opinion	24 (48%) 14 (28%)
**Modified acceptability and credibility measure**		0-10 scale, mean (SD)
9. How logical does this type of review seem to you?	Not logical — very logical	7.83 (2.14)
10. How successful do you think the review will be in changing how you manage your pain?	Not successful — very successful	6.14 (2.72)
11. How confident would you be in recommending this review to a friend?	Not confident — very confident	7.29 (2.82)
12. How much improvement in your ability to manage pain do you think will occur?	No improvement — much improvement	5.44 (2.74)

#### Qualitative evaluation

Further understanding of acceptability of the PROMPPT review was obtained from qualitative interviews with participants (*n* = 15), which investigated reasons behind perceptions of acceptability. Participants talked about aspects of acceptability across all TFA constructs, apart from the domain of opportunity costs after experiencing the PROMPPT review.

#### Global acceptability (how acceptable was PROMPPT overall)

Overall, interview participants appeared to perceive the PROMPPT review as a good idea and something they would recommend to others. They considered it appropriate for the review to be conducted by practice pharmacists as they were knowledgeable about medicines and the review redistributed workload away from overstretched GPs.

#### Affective attitude (feelings and emotions related to PROMPPT)

Interview participants told us they were pleased to be invited to a PROMPPT review and grateful someone was taking an interest. This positive reaction was mixed, with some expressing uncertainty about pharmacists’ qualifications and around what might happen at the review. Some participants reported concerns about their opioids being stopped, and others were hopeful for better pain relief or sceptical of there being any benefits. Participant anxieties appeared to be alleviated by pharmacists during the review and, overall, participants reported finding the experience enjoyable. They felt reassured and pleased they had an opportunity to discuss their pain and pain management, although some said they hoped the review would have offered more for reducing pain.

*I was anxious, irritated, whatever, I thought they’re going to mess with my medication again and I’m okay how I am. And I was sort of anxious and irritated at the same time but after speaking to him I felt more at ease (ID 2288)*

A participant who reduced their opioids was ‘impressed’ and ‘happy’ with the suggestion of making a change. However, one participant reported leaving their review feeling ‘puzzled’ as she thought the pharmacist was there to help and felt ‘fobbed off’, describing a lack of follow-up as being ‘left out in the cold’ and ‘abandoned’.

#### Burden (effort required to participate in PROMPPT)

Participants described little or no effort was required to participate in the review. Overall, appointments were reported to be easy to organise, convenient, and straightforward. Participants said they liked having a 30-min appointment and did not view having a longer than normal consultation as a burden. Discussions were described as ‘straightforward’, but discussing change could be ‘difficult’ for those who were happy with their current pain medicine regimen. Although phone consultations were described as ‘fine’, participants expressed a strong preference for face-to-face consultations, even with the extra effort involved in traveling to their GP surgery. Face-to-face consultations were perceived to help them to establish a trusting relationship, open-up and talk more easily, have honest discussions, and show physically how they move and walk.

*I mean she's never met me so she doesn’t know me so…well we really ought to meet face to face to try and work out a solution (ID3104)*

#### Ethicality (fairness of a proactive PROMPPT review)

Participants agreed that it was fair and right to proactively invite patients with persistent pain to a review, and uptake should be voluntary. Patient safety and medicines optimisation were identified as important reasons for a review. There was variation in how often participants believed a review should happen, from every month to every 2 years, with the majority favouring somewhere between 6 and 12 months. Participants also recommended the possibility of booking a review as health status or concerns required.

*I think it’s quite fair because sometimes you need to be able to talk about how you’re managing with whatever you’re managing with, and if you can get any extra help (ID2288)*

#### Intervention coherence (purpose of PROMPPT review and how it works)

Participants had mixed perceptions about the purpose of the review, which included stopping or reducing opioids, saving the NHS money, offering alternative pain relief medicines, ‘better pain control’, reviewing current pain medicines, and promoting patient safety. Expectations appeared to be largely based on participants’ previous experiences of consultations with healthcare professionals about their pain, and some seemed to hope for ‘an answer’ or a ‘magic pill’ (rather than looking for other ways of living well with pain).

Some participants reported that discussions during the review helped them weigh up the positives and negatives of their pain medicines and think differently about how they used them. Participants reported that someone listening was important, and that it made ‘a massive difference’ to them feeling valued. However, those who left the review with unfulfilled hopes for more effective pain medicines typically disengaged with the PROMPPT review process.

*I thought there was going to be an answer...but on my part because I’ve had so many reviews of various descriptions, I don’t really want anymore (ID2288)*

The relevance of the review was questioned by some who were stable and happy with their current pain medication. Whilst many participants said their understanding of pain was unchanged, there were good examples of increased knowledge (e.g. know not to fear the pain, why pain happens, need to decrease opioid medicines slowly, that monitoring helps). Some participants described new knowledge about the role of specific medicines (e.g. paracetamol), and some described unmet information needs relating to managing pain or changing opioid or non-opioid pain medicines.

*I assumed it was to talk about any medications that I’m on and see whether they were still working for me, not working for me. Er, I assumed that he may, if needs be, reduce some or offer alternatives, that kind of thing, and also give you the time that the GPs sadly don’t have (ID2106)*

#### Self-efficacy (confidence to take part in PROMPPT and make changes to opioids)

Many participants said they were confident in having discussions with the pharmacist, although those with hearing difficulties were less confident over the phone. Others, who were uncertain prior to the review, reported gaining in confidence during the review. Where changes to opioids were agreed, participants’ confidence in implementing these varied. Some seemed happy to make the changes because they ‘trusted’ the pharmacist ‘implicitly’ having previous positive experiences of changing medicines, whereas others appeared more hesitant and wanted additional support*.*

*A little bit worried before the phone call…during the phone call, no. You know, I became more confident, and I’d be very confident to have it again (ID1112)*

#### Perceived effectiveness (effectiveness in reducing opioids)

Participants who reported reductions in opioid dose were generally happy with the change. One participant reported experiencing an improvement in pain, but most reported no change in pain as a result.

*The review has helped to live with pain as the pain it’s been a lot better. I have written to the pharmacist to say thank you for the review, it was really helpful, the pain’s a lot better than what it was. (ID1112)*

No change in pain left some participants saying that the impact of the review was minimal. Some said reducing opioids was neither helpful nor useful to them personally but had potential for others who are new to pain management or not coping well with pain. These findings indicated some misalignment between participant perceptions and intended review outcomes. One participant expressed dissatisfaction with the reduction having experienced withdrawal and self-reinstated their former higher dose suggesting a quicker follow-up by the pharmacist would better support patients.

#### Mixed methods analysis of acceptability

Meta-inferences about acceptability are presented in Table 6. This joint display integrates findings from the acceptability questionnaires and qualitative interviews. There were similarities between data sets in relation to the overall acceptability of the review (global acceptability), the level of burden perceived to take part in the review (burden), and perceptions of the fairness of proactively inviting patients with persistent pain for a PROMPPT review (ethicality). There was dissonance in relation to the TFA domain of opportunity costs, the level of interference with participants’ priorities, with low scores on the acceptability questionnaire item. This might be explained by the difficulty in understanding and responding to the question, with 28% of participants not having an opinion on that question. Qualitative data helped to expand the quantitative findings around participants’ thoughts and feelings about the review (affective attitude), their understanding of the purpose of PROMPPT and how it works (intervention coherence), their confidence in taking part in the review (self-efficacy), and their perceptions of the review’s effectiveness for pain management.

**Table 6:** Statistics-by-theme Joint Display of Participant Acceptability of PROMPPT

PROMPPT acceptability domain	Acceptability questionnaire (*n* = 63)	Qualitative framework summary	Inference
**Global acceptability**	Acceptable or completely acceptable *N* = 45 (90%)	Overall PROMPPT review is a good idea, appropriate, and something to be recommended • Participants reported the review was in no way problematic and not missing anything	Confirmation
**Affective attitude**	Like or strongly like the review *N* = 38 (76%)	Varied emotional responses to the review • Pre-review, some participants were *pleased*, *grateful*, and *happy* with prospect of seeing a pharmacist. Others were *unsure*, *worried*, and *concerned* about the possible outcome of the review and impact on health and wellbeing • Post-review, most participants were *happy* and would recommend the review, but some felt *abandoned* by the GP and were *disappointed* with follow-up	Expansion
**Burden**	Review took no effort at all or little effort *N* = 44 (88%)	Little or no effort required to participate in the review • Benefits of face-to-face consultations outweighed any burden of attending in person • Discussions were straightforward overall, except for some participants who were happy with current pain medicine regime	Confirmation
**Ethicality**	Fair or very fair *N* = 42 (84%)	It is fair and right to proactively invite patients with persistent pain to a review if uptake is voluntary, and patients should also be able to request a review	Confirmation
**Intervention coherence**	Agree or strongly agree with purpose of PROMPPT and how it works *N* = 33 (66%)	Initial perceptions of the purpose of PROMPPT varied • For some, PROMPPT was seen as something different, an opportunity to talk, reflect, think differently about medication use, weigh up positives and negatives, and get reassurance • Some participants hoped to get pain relief as current medicines were not working and were looking for a cure rather than other ways of living well with pain • Relevance of the review for all patients was questioned by some participants. Some felt it was important for ‘people earlier on’ when they had not tried many options. Participants who were stable and happy with their current pain medication were unsure of the benefit • Although there were some examples of increased knowledge, unmet information needs remained after the review	Expansion
**Opportunity costs**	Strongly disagree or disagree there was interference with priorities *n* = 24 (48%) No opinion *n* = 14 (28%)	Booking and attending a PROMPPT review did not impact on other participant priorities	Dissonance
**Self-efficacy**	Confident or very confident to make changes *n* = 34 (68%)	Participants expressed confidence in taking part in the review and having discussions with the pharmacist • Developing a good relationship with the pharmacist supported confidence in the review and any changes suggested • Having previous positive experiences consulting with the pharmacist contributed towards this feeling of trust and confidence • Knowing that pharmacists were qualified and had extra training on medicines contributed towards this feeling of trust and confidence • Confidence in implementing changes varied. Some were happy with making changes, whereas others were more hesitant and expressed a desire for additional support to implement the plan suggesting unmet needs	Expansion
**Perceived effectiveness**	Agree or strongly agree the review will change pain management *n* = 30 (60%)	Few participants anticipated the review’s effectiveness. It was only after experiencing the review that participants could see the potential benefit • Some felt that the review’s goal should not be to remove opioids completely, as there was a place for pain medication to be able to function • Participants were generally happy with the suggested changes. Most reported no change in pain as a result, and one experienced an improvement • Some participants reported other benefits of the review, for example: learning that their current pain medicines were the right ones for them (reassurance & confidence), appreciating having a clinician to discuss their pain management with, gaining knowledge about pain medicines with, and one participant reported being motivated to ‘dig out’ their pain management and CBT booklets and revisit previous strategies • Despite this, some participants felt the impact of the review was minimal. Some felt it was neither helpful nor useful to them personally but had potential for others • Only one expressed dissatisfaction with the suggested reduction having experienced withdrawal and self-reinstated back to his higher dose	Expansion

### Objective 5: Fidelity of intervention delivery

#### Quantitative evaluation

**Fig. 3:**
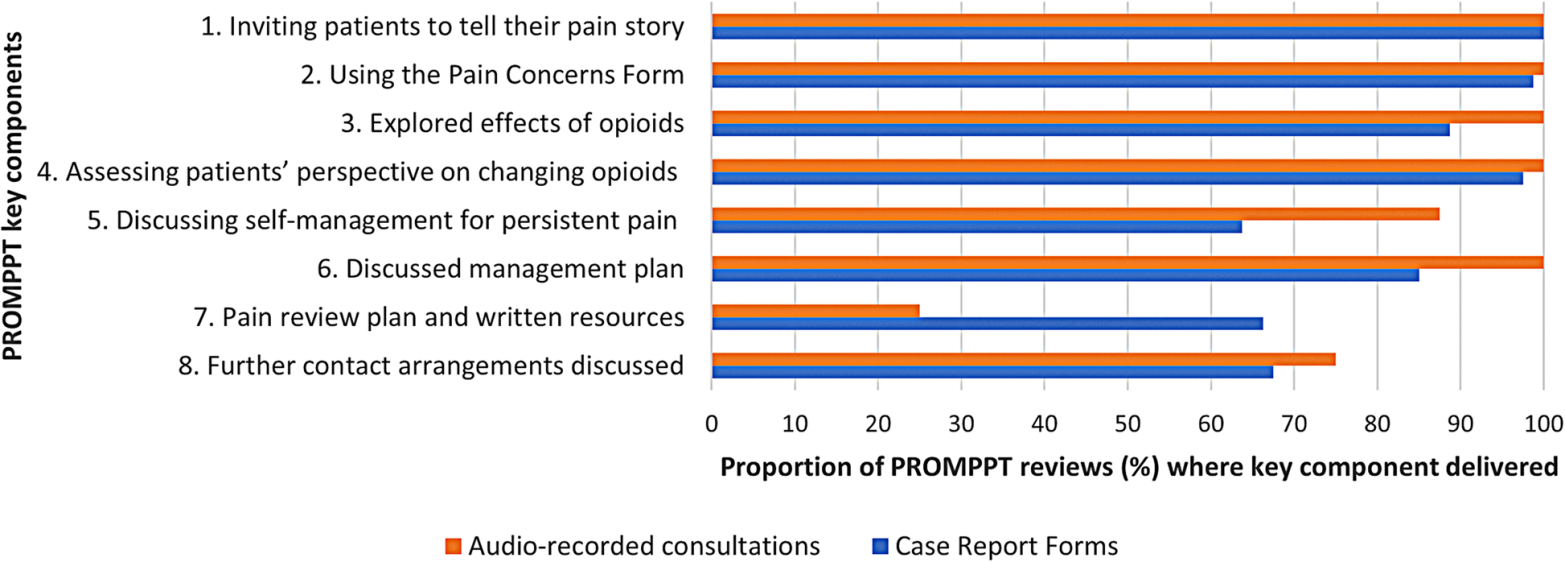
Delivery of key PROMPPT review components from case report forms and audio-recorded consultations

According to pharmacist-completed CRFs, 67 out of 80 PROMPPT reviews (84%) included 6 or more of the key PROMPPT components. Audio-recordings of a sample of reviews demonstrated that nearly all (seven out of eight) included six or more key components. Delivery of each of the pre-specified key components of the PROMPPT review, based on data from both CRFs and audio-recordings, is shown in Fig. 3. Discussing options for self-care, completing the review plan/providing written resources, and making arrangement for further contact were the least frequently delivered components according to both CRFs and audio-recordings. However, it is possible that audio-recordings during the review missed occasions when pharmacists sent the pain review plan and/or links to information resources after the review.

#### Qualitative evaluation

Interview findings relating to each of the key PROMPPT review components are presented below:

##### Inviting participants to tell their pain story

Some participants reported being happy that they ‘managed to discuss everything’ they wanted.

Whatever I wanted to say she sort of gave me the chance to say it, you know. (ID3271)

Other participants said that they did not get the opportunity to discuss their pain as they had expected to.

I would have liked her to at first, ask me about where the pain was, and how I developed it and how long I’ve had it, but she didn’t ask any of those questions at all. (ID3119)

##### Using the pain concerns form 

The majority of participants reported that they could not remember using the Pain Concerns Form during the review.

I can’t remember, I don’t think I did. (ID3104)

##### Exploring the effects of opioids

There was a mixed picture in terms of experiences of discussing the effects of opioids. Some participants described discussing side effects and how the pharmacist asked if they were experiencing any problems with taking opioid medicines and advised that opioids should only be used for ‘short periods’. Others said that side effects and risks were not discussed, were not sure if they were, or could not remember them being discussed. Some participants said they did not know what the possible side effects were or that taking opioid medicines had become so habitual they were used to the side effects or did not associate them with opioids, highlighting some missed opportunities for pharmacists to talk more about side effects and potential harms.

I mean am I going to grow another head. I've still got brown hair at 72, are they really doing any harm? (ID 4093)

##### Assessing the participant’s perspective on changing opioids

In interviews, participants described a range of discussions with the pharmacist about making changes to pain medication. Some participants talked about various reasons why they did not want to make changes to their opioids. However, it was difficult to determine how well pharmacists assessed the participant’s perspective on changing opioids from these interviews.

##### Discussing self-management for persistent pain

Many participants said that self-care and non-drug pain management strategies were not discussed at the review. Furthermore, in the interviews, these participants gave examples of self-management activities they had previously tried, suggesting missed opportunities by the pharmacists to explore their previous experiences and to discuss living well with pain. However, there were some good examples including discussions about exercise, remaining active, sleep, diet, relaxation, and meditation. For example, one participant discussed strategies she had learnt at a previous pain management course:

Yes, I did discuss with him the fact I meditate, and I’m trying to keep moving as well, so even if it’s just walking round and round the kitchen or something, you know, I’m trying to keep mobile even in these difficult times. (ID2106)

##### Agreeing a management plan

Some participants described agreeing changes to their opioid medicines including reducing the number of tablets taken each day, reducing the strength of opioids, and changes to non-opioid medicines. Making changes to opioid and non-opioid medicines were often discussed together, for example increasing paracetamol whilst reducing codeine. When changes had been suggested, some participants described being uncertain about what the next steps were, highlighting potential unmet need for support. Most participants said that they were involved and given a choice during the decision-making process when discussing changes to their medicines.

No, he might say something along the lines of, what do you think about or there is the option, you know, that sort of thing. He never says, we’ve got this, you’re going on it, or anything like that. He’s very good yeah so I’m 100% involved. (ID2106)

However, some described a lack of collaboration with pharmacists already having a plan and not giving them a choice.

##### Providing a pain review plan and information resources

Most participants said that they did not receive a pain review plan or any written information or leaflets after the review. Five participants recalled receiving a plan following the review and were sent it electronically, but one participant said that there were no boxes filled in, and other participants said they had not looked at it yet. One participant had received a video from the pharmacist about exercises but had forgotten about it.

##### Discussing follow-up and further contact arrangements

Whilst some participants had a follow-up booked at the time of the interview, most said they did not remember discussing, or did not discuss, follow-up. When follow-up was discussed or booked, participants reported feeling that they could ring the pharmacist ‘anytime’ they needed to, which reassured them about making changes. Those waiting for follow-up to be arranged reported feeling unsure about how this would happen. Others did not seem satisfied with being advised to ring the pharmacist (e.g. reported they felt abandoned) and said they preferred the pharmacist to arrange follow-up. Participants talked about being unsure if or when they should make an appointment, being unable to get through to the surgery, and perceiving that the pharmacist would be too busy.

I forgot to mention that I had arthritis in more than one place which as I say was my fault completely but erm, I did wonder about getting in touch with her again, but I thought – no, I knew - she was going to be busy, because she did say, I think quite a number of reviews with people over the following weeks after she’d spoken to me. (ID 3073)

Where participants were waiting to be contacted about follow-up, some said they wondered why they had not been contacted and felt they needed follow-up because they wanted to know what the next steps for them were (e.g. carry on with a reduction, reduce it again), ‘add things’ to discuss that they thought about after the first review, discuss changes they had made, or because they needed help with maintaining changes and/or needed repeat prescriptions. Some participants said they did not want any follow-up as they were unsure what the purpose of one would be, wanted to wait a while to try and reduce the medicines before getting back in touch, or felt that there was no point.

If I’m truthful I don’t want to really because I don’t feel like they can do anything else. (ID2288)

#### Mixed methods analysis of fidelity

Meta-inferences about fidelity are presented in Table 7. This joint display integrates findings from the CRFs, audio-recordings, and qualitative interviews. There was dissonance between data sets relating to the degree to which participants were invited to tell their pain story and use of the Pain Concerns Form during the review. Qualitative data helped to expand the quantitative findings about the pharmacist exploring the effects of opioids with the participants during the review and discussions around further contact arrangement following the review. Findings from the different datasets regarding discussions of self-management for persistent pain and providing pain review plans and written resources following the review were congruent.

**Table 7:** Statistics-by-theme Joint Display of Fidelity of PROMPPT Delivery

Key PROMPPT intervention component	Case report form (*n* = 80)	Audio-recorded consultation (*n* = 8)	Qualitative framework summary	Inference
1. Inviting patients to tell their pain story	100% (*n* = 80)	100% (*n* = 8)	Mixed experiences of storytelling • Some participants managed to discuss everything they wanted and felt comfortable sharing their ‘pain story’ • Others did not get the opportunity to discuss pain experience, despite expecting pharmacists to ask them	Dissonance
2. Using the Pain Concerns Form	99% (*n* = 79)	100% (*n* = 8)	Most participants did not recall the Pain Concerns Form being used during the review	Dissonance
3. Exploring the effects of opioids	89% (*n* = 71)	100% (*n* = 8)	Mixed experiences of discussing the effects of opioids • Some described discussions with the pharmacist about side effects and being asked if they were experiencing problems • Others felt that side effects were not discussed, were not sure if they were, or could not remember them being discussed • Some participants did not know what the potential side effects and risks of opioids were, highlighting missed opportunities for pharmacists to talk more about potential harms	Expansion
4. Assessing patients’ perspective on changing opioids	98% (*n* = 78)	88% (*n* = 7)	Evaluating how well pharmacists assessed the patient’s perspective on changing opioids was difficult to determine from the patient interviews	-
5. Discussing self-management for persistent pain	64% (*n* = 51)	75% (*n* = 6)	Many participants felt that self-management, living well with pain, and doing other things to help with their pain were not discussed at the review • Some recalled discussions about exercise, keeping moving, weather, sleep, diet, and relaxation/meditation	Agreement
6. Discussed management plan	85% (*n* = 68)	63% (*n* = 5)	• Participants talked about a range of plans to make changes to their opioid and non-opioid pain medication • Some talked about how they had agreed not to make changes to their opioid medication • Most discussed being actively involved in discussions and decisions around changing their opioids medicines however some described a lack of collaboration with the pharmacist already having a plan	Agreement and expansion
7. Pain review plan and written resources	66% (*n* = 53)	75% (*n* = 6)	• Most participants said that they did not receive a pain review plan or any written information or leaflets after the review • One received a blank pain review plan, whilst others had received it but were yet to look at it or had forgotten about it	Agreement
8. Further contact arrangements discussed	68% (*n* = 54)	88% (*n* = 7)	Experiences of follow-up varied • Some participants had a follow-up booked at the time of the interview and knew they could contact the pharmacist anytime, providing reassurance to them about making changes • Half of patients did not remember discussing, or did not discuss follow-up • Some were waiting for follow-up to be arranged but were not sure how this would happen	Agreement and expansion

## Discussion

The overall goal of this study was to determine the feasibility of conducting a definitive RCT to evaluate a new practice pharmacist-led primary care intervention (PROMPPT), which aims to reduce opioid use by patients with persistent pain, where appropriate, without increasing pain or pain-related interference. To address this goal, our specific objectives were to assess the feasibility of recruitment, retention and data collection for a definitive cluster RCT, uptake and acceptability of the PROMPPT intervention, and fidelity of PROMPPT review delivery by practice pharmacists. Performance against pre-specified progression criteria, where applicable, is outlined in Table 8.

**Table 8:** Performance against pre-specified progression criteria

**Objective**	**Pre-specified progressions criteria**^a^	**Performance**	**Implications for the main trial**
Recruitment to the MOPP questionnaire study and retention at 3-month follow-up	• ≥ 20% of eligible patients consent to participate • ≥ 70% of consenting patients complete a 3-month follow-up questionnaire	• 14.5% (148/1020) of eligible patients consented to participate • 83.1% of MOPP participants completed a 3-month follow-up questionnaire	The recruitment findings informed the sample size estimate for the main trial. As the main loss of potential participants was at the ‘consent to contact’ stage, the recruitment process for the definitive cluster RCT will be changed, removing the ‘consent to contact’ stage and introducing text (SMS) invitations from GP practices for potentially eligible patients
Uptake of the PROMPPT intervention	> 50% of participants invited attend at least one PROMPPT consultation	90.9% of participants invited attended the initial PROMPPT review consultation	The process of GP practices inviting patients to schedule a PROMPPT review remained the same
Acceptability and credibility of the PROMPPT intervention	• Mean acceptability/credibility score ≥ 5 • Evidence from interviews about intervention acceptability	• Mean acceptability/credibility score 6.7 (*SD* 2.2) • Overall, participants interviewed perceived the PROMPPT review as acceptable, easy to organise and participate in, and not burdensome. There was a preference for face-to-face consultations. There were varied emotional responses to the PROMPPT review and varied perceptions of its purpose. Some participants reported feeling worried before the review (about what would happen), and some did not feel it was relevant to them. Following the review, participants seemed more reassured and more satisfied with the plan when clear arrangements for a follow-up appointment had been made	In light of these findings, for the main trial, the option of face-to-face reviews was offered. Patient invitation letters and information leaflets were revised, and the pharmacist training was updated to include new case studies and to emphasise the importance of clear follow-up plans (see Fig. 4)

^a^Proceed to main trial with no more than minor modifications

### Feasibility of study methods and implications for the design of a definitive cluster RCT

The percentage of eligible patients consenting to the MOPP questionnaire study was lower (15%) than the pre-specified success criterion (20%), although retention at 3-month follow-up was higher than the target of 70%. The main loss of potential participants was at the ‘consent to contact’ stage, and, following discussion with our PPI group, we decided to change the recruitment process for the definitive cluster RCT, removing the ‘consent to contact’ stage and introducing text (SMS) invitations using general practices existing systems for bulk messaging for potentially eligible patients with a mobile phone that could receive SMS on the practice record. The study findings were also used to estimate the required sample size for a definitive cluster RCT more precisely. Even with the lower recruitment rate, the sample size calculation for the cluster RCT suggested that it would be feasible to achieve the definitive trial aims by recruiting between 30 and 40 general practices, which was in the range we had been planning for. We discussed the study findings, proposed changes, and re-estimated sample size with the independent Trial Steering Committee (TSC) who were satisfied that we could progress to a main trial.

The proposed co-primary outcomes for a definitive trial were the Brief Pain Inventory (BPI) and reduction in opioid use, measured as reduction in daily MED. Completion rates were high for key self-reported outcome measures, including the BPI, which gives confidence in the use of these measures in a definitive trial. We found that the self-reported pain medicines use questionnaire was suitable for the purpose of calculating daily MED in most cases. After crosschecking incomplete or ambiguous questionnaire entries (drug name and/or strength/dose unit) with the EMR, there was sufficient information to estimate the daily MED for 91% of participants. Acknowledging that self-reported opioid use may be subject to social desirability and recall bias, we also explored the feasibility of measuring opioid use using prescribing data from the EMR, which may give a more objective measure. However, we found that, alone, this was not a reliable source of sufficiently detailed information to permit calculation of daily MED. Furthermore, whilst the EMR provides information on prescriptions issued, it cannot account for patients not taking their medication as prescribed. Good accuracy of self-reported prescribed pain medicines use (including opioids) by people with chronic pain when compared with an administrative prescription claims database has previously been reported [48], and self-reported opioid use has been successfully used in other trials of opioid tapering interventions [8, 49]. Therefore, it was agreed with the independent TSC that change in daily MED should still be the co-primary outcome in the definitive cluster RCT, and, to provide consistency of measurement using one method, MED would be estimated from self-reported pain medicines data, with the option to contact participants where there are incomplete or ambiguous questionnaire entries and, if needed, to obtain missing information about drug strength from the electronic prescribing record. We also discussed study findings relating to the pain medicines use questionnaire with our PPI group, and as a result, reduced the recall period for self-reported opioid use from 4 weeks to 1 week, and made some small changes to the wording and layout of the pain medicines use questionnaire with the aim of making it easier to complete and improving the completeness data collection.

### Acceptability and feasibility of delivering PROMPPT

Uptake of the PROMPPT review was high (91%), and analysis of audio-recorded reviews and CRFs indicated that, in general, PROMPPT reviews were delivered well with most key intervention components delivered as intended. The findings from acceptability questionnaires and qualitative interviews were discussed by the research team. The mean acceptability/credibility score from the questionnaire exceeded the pre-specified success criterion and the responses to the remaining items in the questionnaire, and the qualitative findings from interviews indicated that, overall, the PROMPPT review was acceptable to patient participants. These findings were summarised in the report to the independent TSC, who confirmed that the success criteria for progression to a main trial had been met.

### Implications for refinement of the PROMPPT intervention and training

**Fig. 4:**
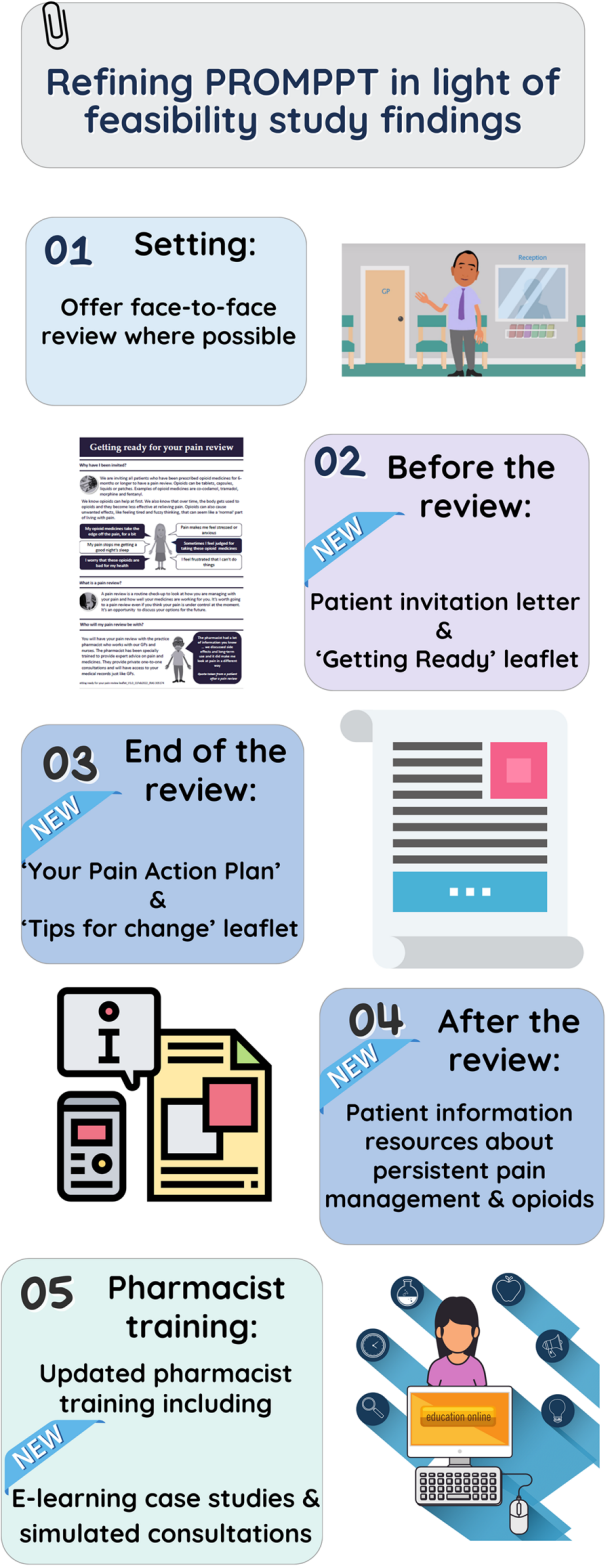
Refining PROMPPT in light of feasibility study findings

The findings also highlighted areas where the PROMPPT intervention and pharmacist training could be improved. Due to the COVID-19 pandemic, most reviews were conducted remotely by telephone, but participants interviewed expressed a preference for face-to-face review in future if possible. Interview findings suggested that refinement of the PROMPPT invitation letter and information sheet was needed, to reduce uncertainty and anxiety about the review and to highlight its potential relevance to all patients prescribed opioids for persistent pain. Audio-recordings and interview findings suggested that changes to the Pain Concerns Form were also needed as this was often not used or seemed to get in the way of the conversation between patient and pharmacist. A PPI workshop discussed these findings. The PPI group felt it was important, where possible, to allow patient choice regarding face-to-face or remote reviews, and both options are offered in the main trial. The PPI group helped refine the PROMPPT review invitation letter and co-produced a new pre-consultation leaflet (replacing the Pain Concerns Form) to help patients identify their priorities for the PROMPPT review, which they named the ‘Getting ready for your pain review’ leaflet. In addition, unmet information needs were described by participants interviewed, and the patient information resources were reviewed and updated to reflect these. Study findings also suggested that changes to the pharmacist training were needed to emphasise the importance of discussing and clearly documenting a management plan so that patients are clear about the next steps and arrangements for follow-up when they leave the review. The pharmacist e-learning was updated accordingly, including the addition of case studies and simulated consultations informed by the audio-recorded consultations and interviews from this study. The main changes made to PROMPPT based on the findings of this study are illustrated in Fig. 4.

### Study strengths and limitations

A key strength of this study is its focus on an intervention that is well-grounded in theory and was designed with a range of stakeholders in line with recommendations for the development of complex interventions [50]. In addition to including patients in the mixed stakeholder group, we were also able to draw on the expertise of a separate PPI group, which has supported the PROMPPT research programme since the pre-funding stage. Members of this group provided a patient perspective on the findings and were invaluable in helping us refine patient-facing documentation. Another strength is that the study was undertaken in a real-world setting using pharmacists who were already working in GP practices. Therefore, the identified facilitators and barriers to delivery identified are likely to represent what would happen if the intervention was implemented in general practices. The PROMPPT review adopts a personalised, shared-decision-making approach, consistent with recent best practice guidance on optimising potentially dependence-forming medicines [51, 52]. Since this work was conceived, structured medication reviews (SMRs) have become a key component of NHS England’s strategy to reduce potentially harmful polypharmacy and reduce inappropriate prescribing of potentially dependence-forming medicines [51], and practice pharmacists are expected to lead on delivering SMRs [53], but there is little specific training currently available regarding SMRs with patients on opioids.

Our study also provides a worked example of good practice in mixed methods data integration during intervention feasibility assessment. First, we included qualitative assessment of acceptability in our study progression criteria as proposed in new recommendations for progression criteria [54]. This novel approach facilitated decision-making about next steps to be based upon all available data, not just quantitative metrics about recruitment and intervention uptake. Second, we merged quantitative and qualitative results in joint displays, in line with new recommendations for mixed methods feasibility studies [55]. This has provided more nuanced understanding and allowed us to make overall inferences, strengthening conclusions made about PROMPPT feasibility. The global assessment of acceptability from both quantitative and qualitative findings aligned, providing confidence in the conclusion that the PROMPPT review is generally acceptable to patient participants. Similarly, mixed methods findings provided a comprehensive understanding of fidelity, particularly identifying issues with the Pain Concerns Form not being used during the review and pain review plans not being completed and given to participants, which required attention prior to main trial.

This study has some limitations. First, because the study was delivered during the COVID-19 pandemic, social distancing measures meant the majority of PROMPPT reviews were delivered remotely by telephone. It is possible that face-to-face PROMPPT reviews in the context of the definitive cluster RCT may highlight different or additional barriers and facilitators that were not identified in this study, and the main trial includes a process evaluation to explore this. Furthermore, although our PPI group continued to support the study remotely during the pandemic, one member was unable to join due to having no internet access. We therefore obtained perspectives on conducting remote consultations from patient representatives who were able and willing to participate in online meetings and acknowledge that other people, who were not able to join online, may have given a different perspective. Finally, we excluded non-English language speakers, which has implications for the generalisability of the findings. Steps were taken, including use of interpreter services, to ensure non-English speakers could be included in the main trial.

## Conclusions

This study provides the first evidence regarding the feasibility of delivering the practice pharmacist-led PROMPPT intervention in general practice. The results of this study indicated that the PROMPPT review is acceptable to patients, and that a definitive cluster RCT was feasible in the target population of patients prescribed opioids for at least 6 months for persistent pain in primary care. The study identified aspects of the intervention, pharmacist training, and study methods where improvements could be made. The recruitment process, patient-facing review documentation, pharmacist training, and data collection tools were refined in light of these findings, ahead of the main cluster RCT to determine the clinical and cost-effectiveness of PROMPPT in reducing opioids use, where appropriate, without increasing pain or pain-related interference.

## Data Availability

The datasets generated and/or analysed during the current study are not publicly available due to consent issues but are available from the corresponding author on reasonable request.

## Abbreviations

BPI: Brief Pain Inventory
CRF: Case report form
CRN: Clinical research network
CTU: Clinical trials unit
EMR: Electronic Medical Record
GP: General practitioner
IQR: Interquartile range
NSAID: Nonsteroidal anti-inflammatory drug
NHS: National Health Service
MED: Morphine equivalent dose
MOPP: Management of Opioids and Persistent Pain study
MRC: Medical Research Council
NIHR: National Institute for Health and Care Research
PPI: Patient and public involvement
PROMPPT: Proactive clinical Review of patients taking Opioid Medicines long term for persistent Pain led by clinical Pharmacists in primary care Teams
RCT: Randomised controlled trial
SD: Standard deviation
SMR: Structured medication review
SMS: Short message service
TSC: Trial Steering Committee
TFA: Theoretical framework of acceptability
UK: United Kingdom
